# High-Mannose But Not Complex-Type Glycosylation of Tetherin Is Required for Restriction of HIV-1 Release

**DOI:** 10.3390/v10010026

**Published:** 2018-01-05

**Authors:** Abdul A. Waheed, Ariana Gitzen, Maya Swiderski, Eric O. Freed

**Affiliations:** Virus-Cell Interaction Section, HIV Dynamics and Replication Program, National Cancer Institute, Bldg. 535, Room 108B, 1050 Boyles St., Frederick, MD 21702-1201, USA; algitzen2@gmail.com (A.G.); swiderskimaya@gmail.com (M.S.); efreed@mail.nih.gov (E.O.F.)

**Keywords:** human immunodeficiency virus, tetherin, mutant, *N*-linked glycosylation, glycosylation inhibitors, cell surface, virus release, tunicamycin, kifunensine

## Abstract

Tetherin is an interferon-inducible antiviral protein that inhibits the release of a broad spectrum of enveloped viruses by retaining virions at the surface of infected cells. While the role of specific tetherin domains in antiviral activity is clearly established, the role of glycosylation in tetherin function is not clear. In this study, we carried out a detailed investigation of this question by using tetherin variants in which one or both sites of *N*-linked glycosylation were mutated (N65A, N92A, and N65,92A), and chemical inhibitors that prevent glycosylation at specific stages of oligosaccharide were added or modified. The single *N*-linked glycosylation mutants, N65A and N92A, efficiently inhibited the release of Vpu-defective human immunodeficiency virus type 1 (HIV-1). In contrast, the non-glycosylated double mutant, N65,92A, lost its ability to block HIV-1 release. The inability of the N65,92A mutant to inhibit HIV-1 release is associated with a lack of cell-surface expression. A role for glycosylation in cell-surface tetherin expression is supported by tunicamycin treatment, which inhibits the first step of *N*-linked glycosylation and impairs both cell-surface expression and antiviral activity. Inhibition of complex-type glycosylation with kifunensine, an inhibitor of the oligosaccharide processing enzyme mannosidase 1, had no effect on either the cell-surface expression or antiviral activity of tetherin. These results demonstrate that high-mannose modification of a single asparagine residue is necessary and sufficient, while complex-type glycosylation is dispensable, for cell-surface tetherin expression and antiviral activity.

## 1. Introduction

The innate immune response is the first line of defense against invading viral pathogens. Mammalian cells encode a large number of incompletely characterized factors that impede virus replication at various stages of the virus replication cycle. These inhibitory, or “restriction”, factors are either expressed constitutively or are induced by type-I interferon (IFN). One such restriction factor that interferes with a late stage of the viral replication cycle is tetherin (also known as bone marrow stromal antigen 2 (BST2), cluster of differentiation 317 (CD317) or HM1.24), which inhibits the release of human immunodeficiency virus type 1 (HIV-1) and is counteracted by the HIV-1 accessory protein Vpu [1,2]. Tetherin is expressed constitutively in terminally differentiated B cells and T cells, and was identified as a marker for bone marrow stromal cells and some cancer cells [3,4,5,6,7,8,9,10,11,12,13]. An ~180 amino acid, type II integral membrane glycoprotein, tetherin is localized in lipid rafts at the cell surface and on intracellular membranes [10,14]. Tetherin is a homodimeric glycoprotein that contains a short, *N*-terminal cytoplasmic tail (CT), a transmembrane (TM) domain, a rod-like coiled-coil (CC) ectodomain, and a *C*-terminal glycosylphosphatidylinositol (GPI) anchor [10,14,15]. The CT of tetherin contains an “STS” sequence that is implicated in ubiquitylation, and a highly-conserved tyrosine-based motif, “YxxY”, that is essential for clathrin-dependent endocytosis of tetherin, and activation of nuclear factor-κB (NF-κB) [16,17,18,19]. The TM and GPI anchor provide two membrane anchors that enable tetherin to tether virions with one domain in the viral envelope and the other in the plasma membrane (PM) of the host cell. The specific sequence of the TM domain is implicated in the interaction between tetherin and its antagonists such as Vpu. The ectodomain of human tetherin contains three Cys residues that are required for the formation of homodimers [15,20]. Du Pont et al. recently demonstrated that disulfide bonds are not required for maintaining the coiled-coil or dimeric structure of tetherin, however, dimerization is necessary for tetherin’s antiviral activity [21]. The ectodomain also contains two Asn residues at positions 65 and 92 that are post-translationally modified with *N*-linked oligosaccharides [14,15,20,22]. Deletion of the CT, TM, CC, or GPI domains, or mutating the Cys residues that are required for protein dimerization, abrogates the ability of tetherin to inhibit virus release [1,15,20].

Tetherin inhibits the release of not only HIV-1 but also that of a broad spectrum of other enveloped viruses, including alphaviruses, filoviruses, rhabdoviruses, arenaviruses, herpesviruses, paramyxoviruses, flaviviruses, orthohepadnaviruses, orthomyxoviruses, and other retroviruses (reviewed in [23,24,25]). Many of these viruses have evolved diverse strategies to antagonize tetherin. HIV-1 Vpu counteracts human tetherin by reducing its cell-surface expression via lysosomal and proteasomal degradation [26,27,28,29,30,31,32,33,34] or by trapping tetherin intracellularly and/or preventing its recycling back to the PM [1,2,32,35,36,37,38]. The specificity of HIV-1 Vpu towards tetherin antagonism also varies from species to species. HIV-1 Vpu counteracts tetherins from human, chimpanzee, and gorilla; however, it is relatively inactive against tetherin from other non-human primates and non-primates [28,39,40,41,42]. Simian immunodeficiency virus (SIV) and HIV-2, which do not encode a *vpu* gene, antagonize tetherin through their Nef and envelope (Env) proteins, respectively, in part through an intracellular sequestration mechanism [29,39,42,43,44,45,46,47,48].

As mentioned above, human tetherin contains two putative *N*-linked glycosylation sites in the extracellular CC domain. The requirement for tetherin glycosylation in the inhibition of virus release remains controversial. Early studies reported that *N*-linked glycosylation of Asn 65 (N65) and 92 (N92) is important for the inhibition of HIV-1 release [15,49,50,51,52,53,54]. However, other studies found that mutating N65 and N92 had a negligible effect on tetherin restriction of HIV-1 release [20,55,56]. Some studies reported a correlation between tetherin restriction of virus release and tetherin cell-surface expression [15,50], while others did not observe this correlation [20,53,54,57]. While some studies were carried out with human tetherin, others used tetherins from other species. Studies also differed in their use of viral system to examine tetherin restriction; some studies were carried out with HIV-1 [15,20,50,53,54] whereas others used feline immunodeficiency virus [49,54], prototypical foamy virus [56], xenotropic murine leukemia virus-related virus [51], bovine leukemia virus [52], severe acute respiratory syndrome (SARS)-Coronavirus [57], Lassa fever virus, and Marburg viruses [55].

Asn-linked (*N*-linked) glycosylation is a highly regulated, post-translational modification that is important for the structure and function of eukaryotic proteins. *N*-linked glycosylation is initiated in the lumen of the endoplasmic reticulum (ER) with the cotranslational transfer of a high-mannose oligosaccharide moiety to Asn residues in Asn-X-Ser/Thr motifs (where X is any amino acid except Pro) in the target protein. Trimming of the high-mannose side chains occurs in the ER and Golgi apparatus, and additional sugars (e.g., galactose, fucose, and sialic acid) are attached to generate complex side chains. The glycoprotein is then exported to the PM through the secretory pathway [58]. When transiently overexpressed in 293T cells, several forms of tetherin are detected: the non-glycosylated 23-kDa species, and forms bearing high-mannose modification on either (≈24.5 kDa) or both (≈26 kDa) Asn 65 and 92, and complex-type carbohydrates at either or both positions (≈32 to 40 kDa) [1,15,34,59].

In this study, we carried out a detailed investigation of whether tetherin glycosylation is essential for its ability to inhibit HIV-1 release. We employed glycosylation-site tetherin mutants (N65A, N92A, and N65,92A) and chemical inhibitors of the glycosylation pathway. We observed that oligosaccharide modification of one of the two sites of tetherin *N*-linked glycosylation is necessary and sufficient for tetherin-mediated restriction of HIV-1 release, while complex-type glycosylation is dispensable for tetherin antiviral function.

## 2. Materials and Methods

### 2.1. Plasmids, Antibodies, and Chemicals

The full-length, infectious HIV-1 molecular clone pNL4-3 and the Vpu-defective counterparts pNL4-3delVpu and pNL4-3Udel have been described previously [60,61,62]. pNL4-3delVpu and pNL4-3Udel were kindly provided by K. Strebel (National Institutes of Health (NIH), Bethesda, MD, USA). Vectors expressing tetherin derivatives bearing an *N*-terminal hemagglutinin (HA) epitope tag or an HA tag inserted at residue 154 in the extracellular CC domain of tetherin have been described previously [1,15] and were generously provided by P. Bieniasz (The Rockefeller University, New York, NY, USA). PCR-based mutagenesis was used to introduce Ala substitutions at either one or both of two Asn residues (N65A, N92A, and N65,92A) that are targets for *N*-linked glycosylation. Anti-HA antiserum, kifunensine, and tunicamycin were purchased from Sigma (St. Louis, MO, USA). Alexa Fluor 488 or 594-conjugated secondary antibodies were from Invitrogen (Grand Island, NY, USA). Anti-Vpu, anti-tetherin, and anti-HIV-1 immunoglobulins (Ig) were obtained from the NIH AIDS Research and Reference Reagent Program.

### 2.2. Cell Culture and Transfection

HeLa and 293T cell lines were maintained in Dulbecco-modified Eagle’s medium (DMEM) containing 5% or 10% fetal bovine serum (FBS), respectively. One day after plating, cells were transfected with appropriate plasmid DNA using Lipofectamine 2000 (Invitrogen Corp., Carlsbad, CA, USA) according to the manufacturer’s recommendations. Eight hours after transfection, cells were either untreated or treated overnight with tunicamycin or kifunensine; cells and virus were harvested 24-h post transfection and used for further analysis. To knock down tetherin expression in HeLa cells, one day after plating HeLa cells were treated with 100 nM tetherin small interfering RNA (siRNA) (from Dharmacon, Lafayette, CO, USA) with Oligofectamine transfection reagent (Invitrogen) for 24 h.

### 2.3. Western Blotting Analysis

HeLa or 293T cells were transfected with HIV-1 molecular clones, virions were collected after 24 h, pelleted in an ultracentrifuge, cell and virus pellet were lysed in a buffer containing 50 mM Tris-HCl (pH 7.4), 150 mM NaCl, 1 mM EDTA, 0.5% Triton X-100, and protease inhibitor cocktail (Roche Life Sciences, Basel, Switzerland). After denaturation by boiling in sample buffer, proteins were subjected to sodium dodecyl sulfate polyacrylamide gel electrophoresis (SDS-PAGE), transferred to polyvinylidene difluoride (PVDF) membrane, and incubated with appropriate antibodies as described in the text. Membranes were then incubated with horseradish peroxidase (HRP)-conjugated secondary antibodies, and chemiluminescence signal was detected by using West Pico or West Femto Chemiluminescence Reagent (Thermo Fisher Scientific, Waltham, MA, USA). The protein bands were quantified by using Imagelab-Chemidoc (Bio-rad Laboratories, Marnes-la-Coquette, France).

### 2.4. Virus Release Assays

One day after plating, HeLa or 293T cells were transfected with wild-type (WT) or Vpu-defective pNL4-3 molecular clones; one-day post transfection, virions were pelleted in an ultracentrifuge and cell and virus pellets were lysed [63]. Viral proteins in cell and virus lysates were immunoblotted with HIV-Ig [59] and virus release efficiency was calculated as the amount of virion-associated p24 as a fraction of total (cell-associated p24 and Pr55 plus virion-associated p24) Gag.

### 2.5. Flow Cytometry

293T cells were transfected with WT or mutant human tetherin expression vectors in which an HA tag was inserted in the CC extracellular domain of tetherin and green fluorescent protein (GFP) expression plasmid. HeLa and transfected 293T cells were either untreated or treated with glycosylation inhibitors tunicamycin or kifunensine. Twenty-four hours after transfection or treatment with inhibitors, cells were harvested by adding a solution of 5 mM EDTA in phosphate buffered saline (PBS) and washed in ice-cold 1% bovine serum albumin (BSA)-PBS. The cells were then incubated with anti-HA (mouse) or anti-tetherin (rabbit) antiserum in 1% BSA-PBS for 1 h at 4 °C. The cells were then washed twice in 1% BSA-PBS and stained with Alexa Fluor 594-conjugated anti-mouse (293T cells) or Alexa Fluor 488-conjugated anti-rabbit (HeLa cells) IgG secondary antibody in 1% BSA-PBS for 1 h at 4 °C. The cells were washed three times with PBS, fixed in 1% paraformaldehyde and 5000 gated events were collected using a Becton Dickinson fluorescence-activated cell sorting (FACS) Calibur flow cytometer (BD Biosciences, Hampton, NH, USA). Analysis was performed using FlowJo (TreeStar, Palo Alto, CA, USA).

### 2.6. Immunofluorescence Microscopy

For microscopy studies, HeLa cells were cultured in chamber slides. One day after plating, cells were untreated or treated with kifunensine or transfected with siRNA targeting tetherin. After 24 h, cells were rinsed with PBS and fixed in 3.7% paraformaldehyde in PBS for 30 min. The cells were rinsed with PBS three times, blocked with 3% BSA-PBS for 30 min, and incubated with anti-tetherin antibodies appropriately diluted in 3% BSA-PBS for 1 h. The cells were washed with PBS three times and then incubated with secondary antibody conjugated with Alexa Fluor 488 diluted in 3% BSA-PBS. After washing with PBS three times, cells were mounted with Vectashield mounting media with 4′,6-diamidino-2-phenylindole (DAPI) (Vector Laboratories, Burlingame, CA, USA) and examined with a Delta-Vision RT deconvolution microscope (GE Healthcare Life Scienes, Pittsburg, PA, USA).

## 3. Results

### 3.1. Glycosylation of At Least One Asn Is Required for Tetherin Antiviral Activity

**Figure 1:**
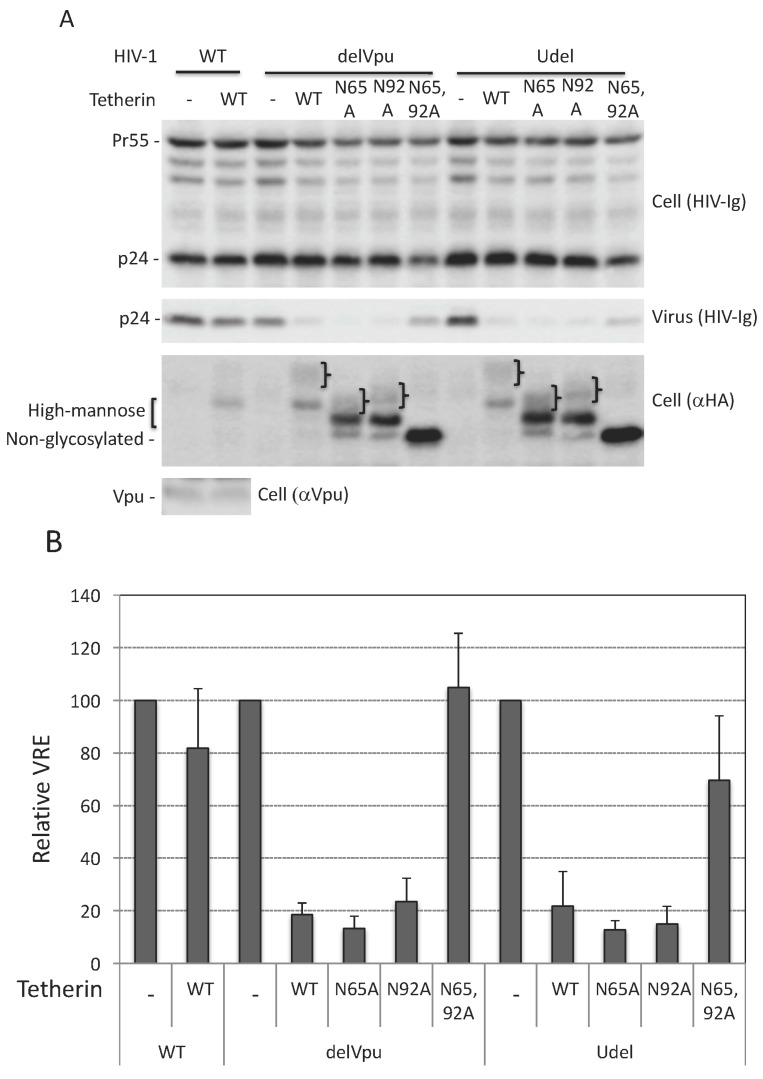

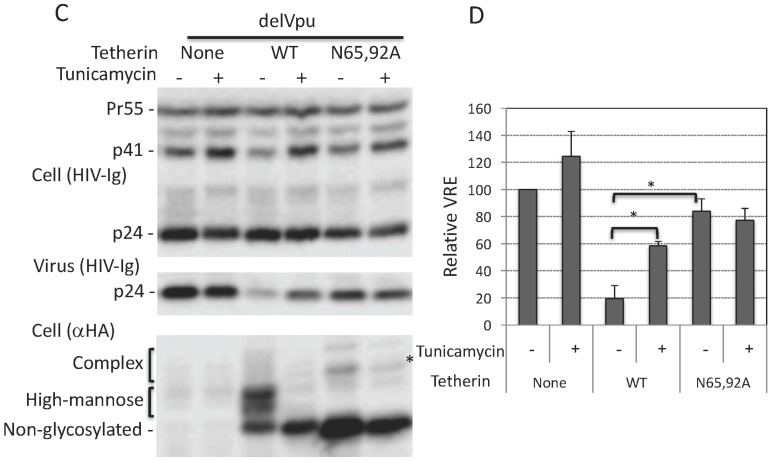
Glycosylation of at least one Asn is required for tetherin antiviral activity. (**A**) 293T cells were transfected with wild-type (WT) or Vpu-defective (delVpu or Udel) pNL4-3 HIV-1 molecular clones, and vectors expressing hemagglutinin (HA)-tagged WT or glycosylation-defective tetherin mutants (N65A, N92A, and N65,92A). One day post transfection, cell and viral lysates were prepared and subjected to western blot analysis with HIV-Ig to detect the Gag precursor Pr55Gag (Pr55) and the capsid (CA) protein p24, anti-HA to detect HA-tagged tetherin or anti-Vpu antisera. The location of non-glycosylated, high-mannose and complex-glycosylated tetherin species is indicated. “}” indicates the position of complex-glycosylated tetherin; (**B**) virus release efficiency (VRE) was calculated as the amount of virion-associated p24 (CA) relative to total Gag in cell and virus. VRE was set to 100% for WT HIV-1 in the absence of tetherin; (**C**) 293T cells were transfected with the delVpu HIV-1 molecular clone with or without HA-tagged WT or N65,92A tetherin expression vector. Eight hours post transfection, cells were untreated or treated with 1 μg/mL tunicamycin for one day, and cell and viral lysates were collected as in Figure 1A. * denotes putative dimeric tetherin; (**D**) VRE was calculated as in Figure 1B; VRE for no tetherin and untreated controls was set to 100%; (**B**,**D**) Data shown are ± standard deviation (SD) from three independent experiments. *p* values (two-tailed paired *t*-test): * *p* < 0.001.

As discussed in the Introduction, the requirement for *N*-linked glycosylation in tetherin antiviral function remains to be clearly established. To define the role of *N*-linked glycosylation of human tetherin in HIV-1 release we used two molecular clones that lack a functional Vpu: a Vpu-deletion mutant pNL4-3Udel [61], and pNL4-3delVpu [62], which has a stop codon at amino acid residue 35 of Vpu. We transfected 293T cells, which do not express endogenous tetherin, with WT and Vpu-defective HIV-1 and analyzed virus release in the presence of WT and glycosylation-defective single (N65A or N92A) or double (N65,92A) mutants of human tetherin. As shown in Figure 1, WT human tetherin inhibited the release of both delVpu and Udel HIV-1 by five-fold, whereas the release of WT (Vpu+) HIV-1 was not significantly reduced under the same conditions. Vpu expression reduced tetherin levels (compare Figure 1 lanes 2, 4, and 9) as reported previously [26,28,29,30,31,34]. The double mutant, N65,92A, was unable to inhibit HIV-1 release (quantified in Figure 1B). This lack of inhibitory activity was not due to reduced expression levels of the N65,92A mutant, as this mutant was expressed at higher levels than WT or the single Asn mutants (Figure 1A). The glycosylation of tetherin does not appear to be critical for its stability, as both single and double mutants are expressed at least as well as the WT. As reported previously [15,20], the single mutants, N65A and N92A, inhibited the release of both delVpu and Udel particles to a similar extent as WT tetherin (Figure 1B). These studies indicate that glycosylation of at least one Asn of human tetherin is necessary and sufficient for the inhibition of HIV-1 release under these conditions.

### 3.2. Tunicamycin Abrogates the Antiviral Activity of Tetherin

To further investigate the role of glycosylation in tetherin function, we overexpressed WT and N65,92A tetherin in 293T cells and treated the cells with tunicamycin, a nucleoside antibiotic that specifically inhibits the first step of *N*-linked glycosylation by blocking the transfer of *N*-acetylglucosamine-1-phosphate to dolichol phosphate [64]. As shown in Figure 1C, treating cells with tunicamycin resulted in a complete loss of glycosylation of WT tetherin, as demonstrated by the observation that the mobility of this tetherin species matched that of the non-glycosylated double mutant (N65,92A). Consistent with the above data with the glycosylation-site mutants, the complete loss of glycosylation imposed by tunicamycin treatment had little effect on tetherin expression, confirming that unlike some other glycoproteins, such as HIV-1 Env [65], tetherin stability does not require glycosylation. Interestingly, tunicamycin treatment significantly disrupted the ability of WT tetherin to inhibit the release of Vpu-defective HIV-1 (Figure 1C,D). The 3-fold increase in virus release upon tunicamycin treatment was similar to that observed upon mutating both N65 and N92 (Figure 1C,D). Tunicamycin treatment had no effect on virus release in the presence of the non-glycosylated tetherin mutant N65,92A. These results support the conclusion that glycosylation of tetherin is necessary for its ability to inhibit HIV-1 release.

### 3.3. Lack of Virus Release Inhibition by N65,92A Tetherin is Linked to Impaired Cell Surface Expression

**Figure 2:**
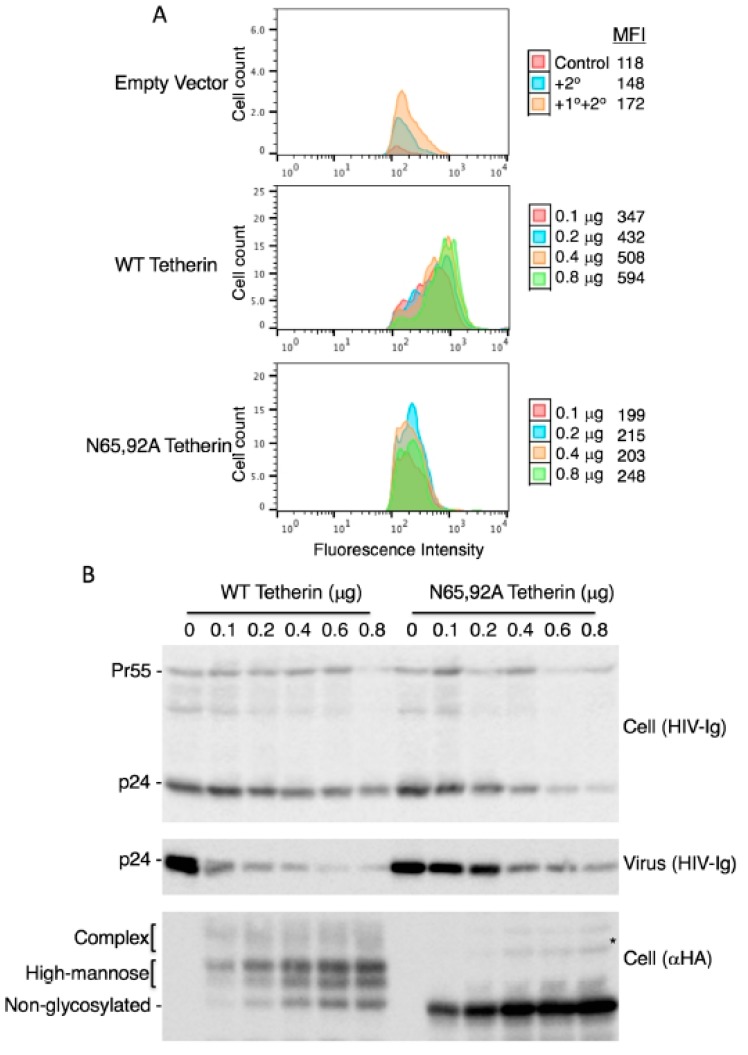

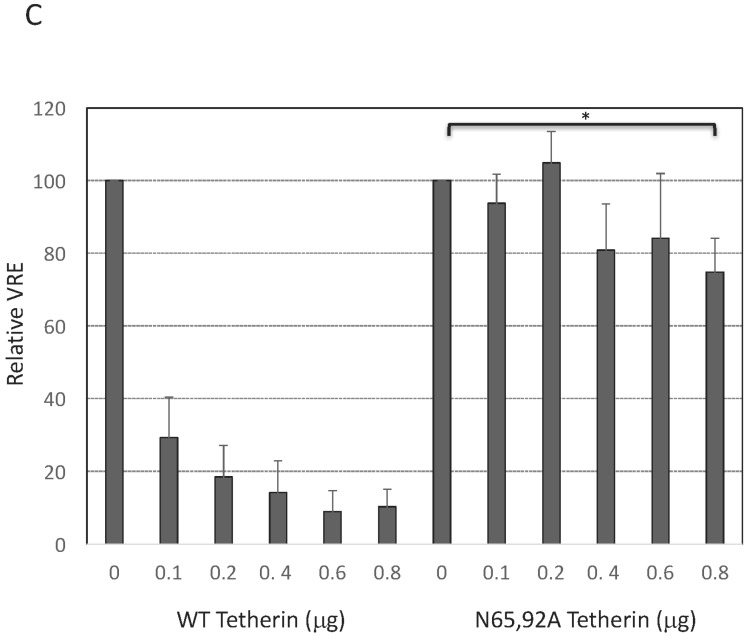
Lack of antiviral activity of N65,92A tetherin is associated with impaired cell-surface expression. (**A**) 293T cells were transfected with the delVpu HIV-1 molecular clone, and increasing amounts (0.1 to 0.8 μg) of HA-tagged WT or N65,92A tetherin expression vector, and 0.1 μg of GFP expression vector. One day post transfection cells were washed, harvested, untreated (control), or treated with anti-HA primary antibody and the Alexa Fluor 594-conjugated secondary antibody (+1°+2°), or treated with secondary antibody alone (+2°), and processed for flow cytometry as detailed in the Materials and Methods Section. Cell-surface expression of tetherin was analyzed with a Becton Dickinson FACS Calibur flow cytometer (BD Biosciences, Hampton, NH, USA) by collecting 5000 gated events; the number of events (cell count) is shown on the *y*-axis. Flow cytometry histograms shown are representative of three experiments. MFI, mean fluorescence intensity; (**B**) 293T cells were co-transfected with the pNL4-3delVpu HIV-1 molecular clone and tetherin expression vector as in Figure 2A. One day post transfection, cells and virus were harvested and subjected to western blot analysis with HIV-Ig and anti-HA antisera as in Figure 1A. * denotes putative dimeric tetherin; (**C**) Virus release efficiency was calculated as in Figure 1B; VRE in the absence of tetherin was set to 100%. Data shown are ± SD from five independent experiments. *p* values (two-tailed paired *t*-test): * *p* < 0.05.

As discussed earlier, there are conflicting reports in the literature on the surface expression of glycosylation-defective tetherin mutants. Some authors reported that the cell-surface expression of the N65,92A mutant was significantly reduced [15,20], slightly reduced [54,57], or not reduced [53] relative to that of WT tetherin. To measure the cell-surface expression of the glycosylation-defective mutant, we expressed WT and N65,92A tetherin in which the HA tag was inserted at residue 154 in the extracellular CC domain and stained with anti-HA antibody as detailed in the Materials and Methods Section. The N65,92A double mutant tetherin was expressed at lower levels on the cell surface relative to the WT (Figure 2A). To increase the cell-surface expression of mutant tetherin, we transfected 293T cells with increasing amounts of WT and N65,92A tetherin expression vector. Although there was a proportional increase in the total cellular expression of tetherin with increasing amounts of transfected DNA for both WT and N65,92A tetherin (Figure 2B), the median fluorescence intensity of mutant tetherin was not increased proportionally with the increasing amounts of the double mutant. This observation supports the conclusion that the N65,92A tetherin is defective for cell-surface expression (Figure 2A). Even at 0.8 μg of N65,92A tetherin expression vector input, the cell-surface expression was lower than that achieved upon transfection of 0.1 μg WT tetherin expression vector. There was no significant decrease in virus release efficiency (VRE) at 0.6 μg input of the N65,92A expression vector; however, there was a small (25%) decrease in VRE at 0.8 μg of input DNA (Figure 2B,C). In contrast, there was a marked and proportional decrease in VRE with increasing amounts of WT tetherin (30% at 0.1 μg and 9% at 0.6 μg of DNA). These results demonstrate that glycosylation is required for proper transport of tetherin to the cell surface and for its ability to inhibit virus release.

### 3.4. Complex-Type Glycosylation Is Dispensable for Tetherin Restriction of Virus Release

**Figure 3:**
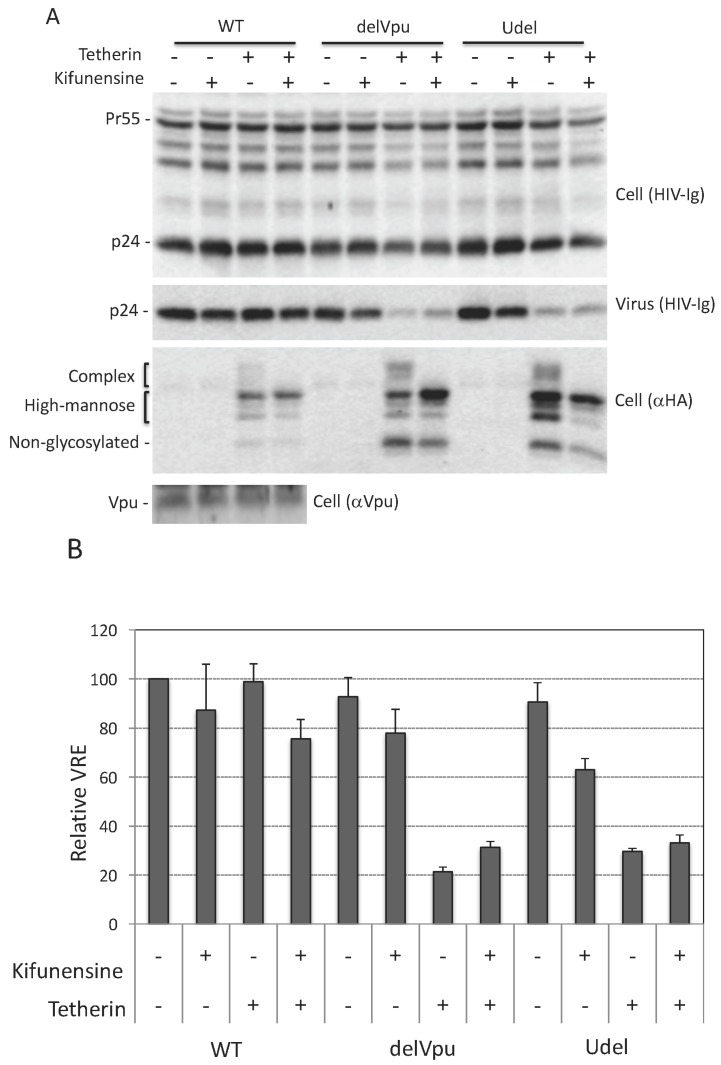

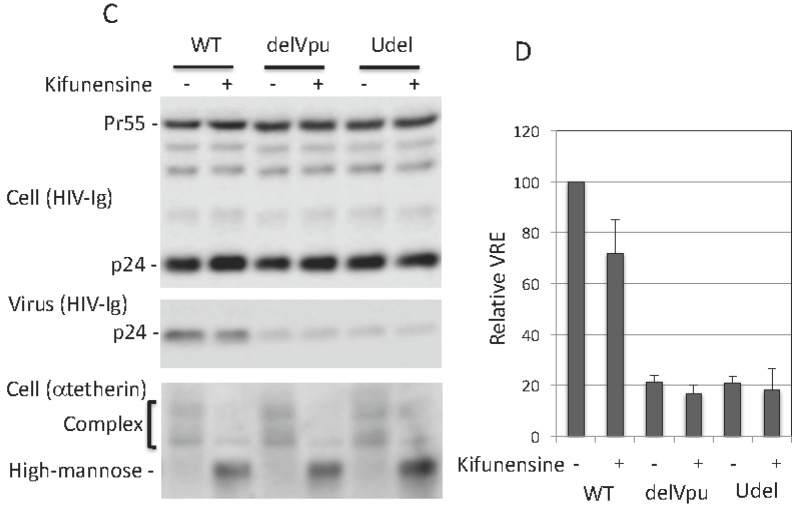
Complex-type glycosylation is dispensable for tetherin restriction. (**A**) 293T cells were transfected with WT, delVpu or Udel pNL4-3 HIV-1 molecular clones, and vectors expressing HA-tagged WT tetherin. Eight hours post transfection, cells were untreated or treated with 10 μM kifunensine for 24 h, and cell and viral lysates were collected and subjected to western blot analysis with HIV-Ig, anti-HA or anti-Vpu antisera as in Figure 1A; (**B**) Virus release efficiency was calculated as in Figure 1B; VRE for WT HIV-1 in the absence of tetherin and kifunensine treatment was set to 100%; (**C**) HeLa cells were transfected with WT, delVpu or Udel pNL4-3 HIV-1 molecular clones, 8 h post transfection cells were untreated or treated with 10 μM kifunensine. One day post treatment cell and viral lysates were collected and subjected to western blot analysis with HIV-Ig, or anti-tetherin antisera as in Figure 1A; (**D**) VRE was calculated as in Figure 1B; VRE for WT HIV-1 in the absence of kifunensine treatment was set to 100%; (**B**,**D**) Data shown are ± SD from three independent experiments.

As discussed in Introduction, tetherin is expressed in several forms: a 23-kDa, non-glycosylated species, and species containing a single high-mannose side chain at either Asn 65 or 92 (≈24.5 kDa), high-mannose side chains at both Asn residues (≈26 kDa), or complex-type side chains at either or both positions (≈32 to 40 kDa) (Figure 1A). Next, we asked whether complex-type glycosylation of tetherin is necessary for its inhibitory activity. To answer this question, we utilized kifunensine, an alkaloid compound that inhibits the activity of ER-associated mannosidase I, an enzyme that is required for trimming and conversion of high-mannose to complex-type side chains [66]. When cells were treated with kifunensine, there was a loss of complex-type glycosylated tetherin, demonstrating that the compound is active (Figure 3A). Despite the loss of complex-type oligosaccharide modifications, kifunensine treatment had little or no effect on the ability of tetherin to inhibit the release of Vpu-defective HIV-1 (Figure 3A,B). The above experiment was carried out by overexpressing tetherin in 293T cells. We also tested the effect of kifunensine on endogenous tetherin in HeLa cells and again observed that kifunensine treatment had no effect on the inhibitory activity of tetherin (Figure 3C,D). As expected, kifunensine treatment shifted the endogenous tetherin from complex-type to high-mannose-modified species (Figure 3C). These results demonstrate that complex-type glycosylation is dispensable for tetherin inhibition of HIV-1 release in the context of both endogenously and exogenously expressed protein.

### 3.5. Complex-Type Glycosylation of Tetherin Is Not Required for Its Cell-Surface Expression

**Figure 4:**
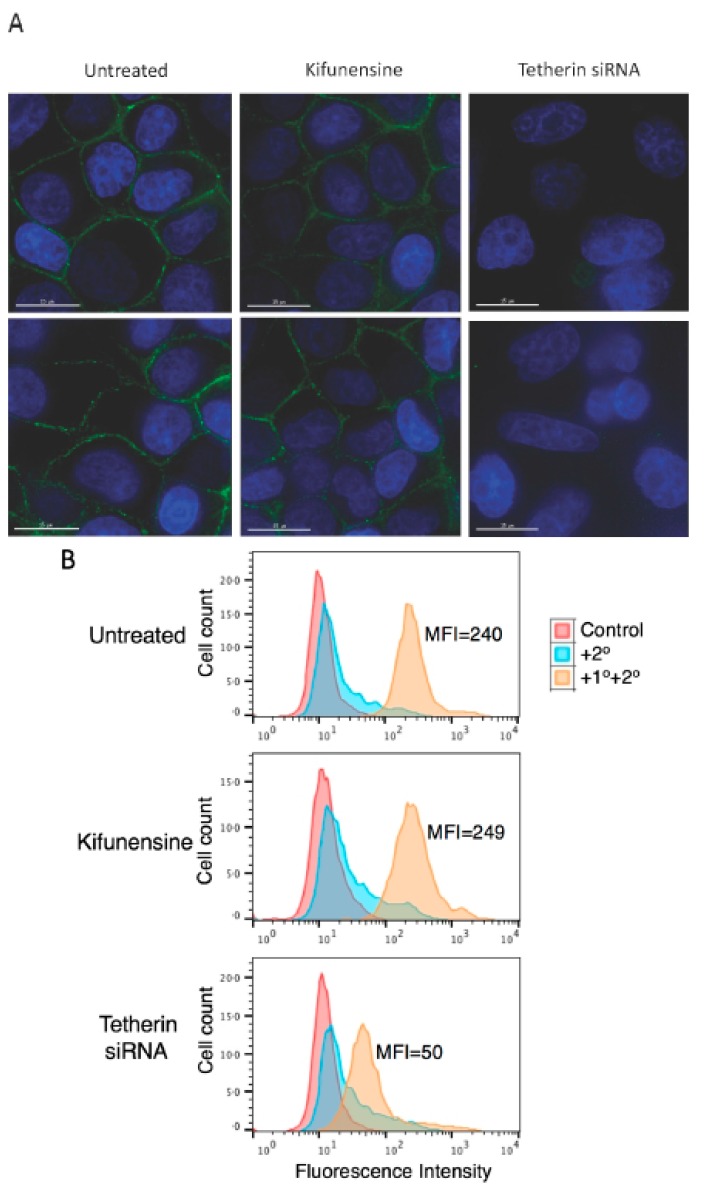
Complex-type glycosylation of tetherin is dispensable for tetherin cell-surface expression. (**A**) HeLa cells were plated in eight-well chamber slides; one day after plating cells were either treated with small interfering RNA (siRNA) to knock-down tetherin expression or treated with 10 μM kifunensine for 24 h. Cells were fixed, stained with anti-tetherin primary antibodies followed by the Alexa Fluor 488-conjugated secondary antibody as detailed in the Materials and Methods Section, and were examined with a Delta-Vision RT deconvolution microscope (GE Healthcare Life Scienes, Pittsburg, PA, USA). Tetherin staining is shown in green and 4′,6-diamidino-2-phenylindole (DAPI) in blue. Scale bars represent 15 μm; (**B**) HeLa cells in 12-well plates were treated as in Figure 4A, cells were harvested and stained with anti-tetherin antisera followed by Alexa Fluor 488-conjugated secondary antibody (+1°+2°) or were stained with secondary antibody alone (+2°) and analyzed with a Becton Dickinson FACS Calibur flow cytometer as in Figure 2A. The number of events (cell count) is shown on the *y*-axis. MFI, mean fluorescence intensity; (**A**,**B**) Representative data from three independent experiments are shown.

The above results demonstrate that complex-type glycosylation of tetherin is not required for its inhibitory function. Since cell-surface expression of tetherin is necessary for inhibition of virus release, these observations would suggest that complex-type oligosaccharide modifications are not required for cell-surface tetherin expression. To directly examine this question, HeLa cells were treated with kifunensine for 24 h and tested for cell-surface expression of endogenous tetherin by both microscopy and flow cytometry. As shown in Figure 4A, immunofluorescence microscopy suggested that kifunensine treatment had little or no effect on the cell-surface expression of endogenous tetherin in HeLa cells. As a control, we knocked-down tetherin expression using siRNA, and as expected we observed a complete loss of cell-surface expression of tetherin. The knock-down of tetherin in siRNA-treated HeLa cells was more than 90%, as determined by quantitative western blotting (data not shown). Flow cytometry analysis confirmed that the cell-surface expression of tetherin in HeLa cells was not diminished by kifunensine treatment, whereas knock-down of tetherin markedly reduced the cell-surface expression (Figure 4B).

**Figure 5:**
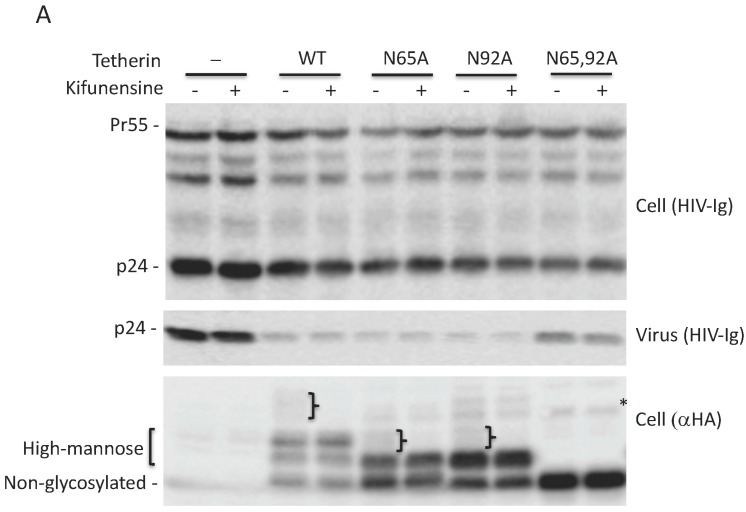

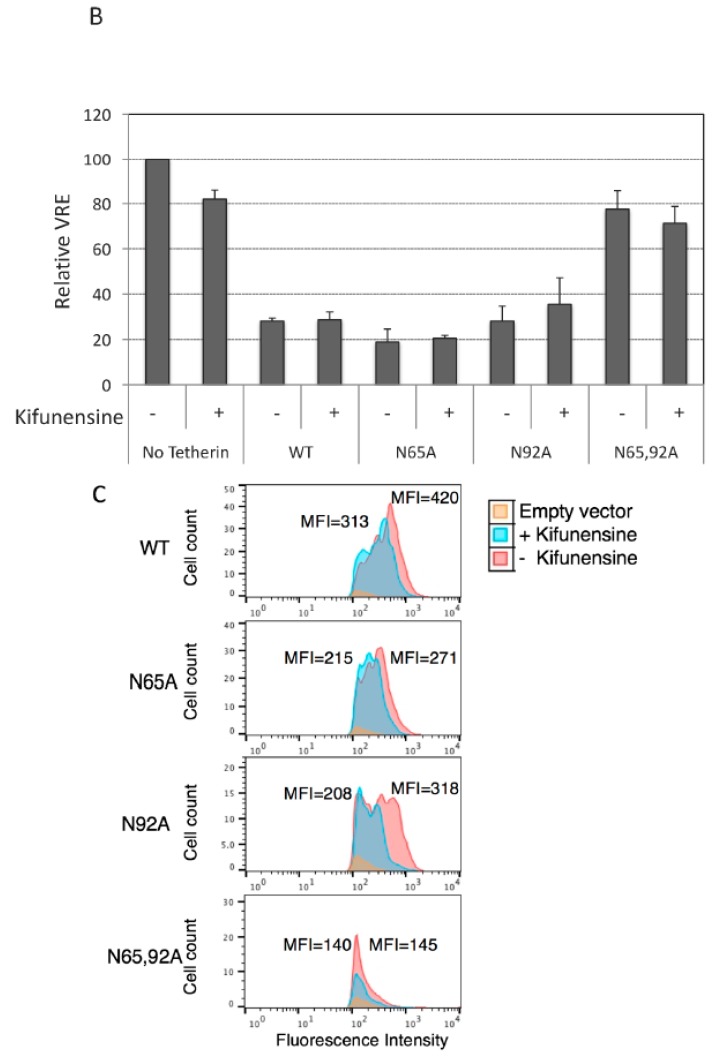

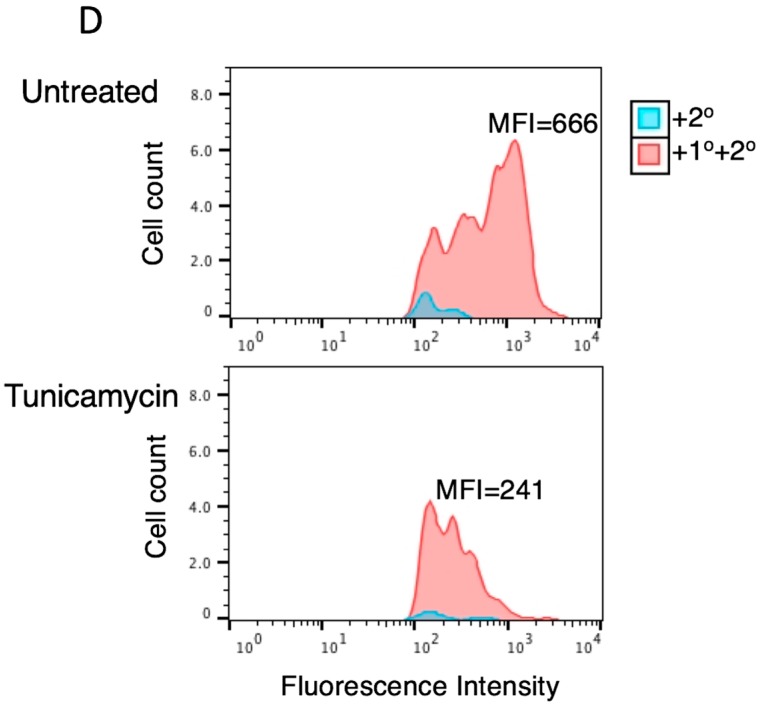
High-mannose glycosylation of a single Asn is sufficient for cell-surface expression of tetherin. (**A**) 293T cells were transfected with the delVpu HIV-1 molecular clone, and vectors expressing HA-tagged WT or glycosylation-defective tetherin mutants (N65A, N92A, and N65,92A). Eight hours post transfection, cells were untreated or treated with 10 μM kifunensine as in Figure 3A, and cell and viral lysates were collected and subjected to western blot analysis with HIV-Ig, or anti-HA antisera as in Figure 1A. “}” indicates the position of complex-glycosylated tetherin. * denotes putative dimeric tetherin; (**B**) Virus release efficiency (VRE) was calculated as in Figure 1B; VRE in the absence of tetherin and without kifunensine treatment was set to 100%. Data shown are ± SD from three independent experiments; (**C**) 293T cells were transfected with vectors expressing HA-tagged WT or glycosylation-defective tetherin mutants (N65A, N92A, and N65,92A) and 0.1 μg of a GFP expression vector. Eight hours post transfection, cells were untreated or treated with kifunensine for 24 h, washed, harvested, and stained with anti-HA primary antibody and Alexa Fluor 594-conjugated secondary antibody and processed for flow cytometry as in Figure 2A; (**D**) 293T cells were transfected with 0.8 μg HA-tagged WT tetherin expression vector and 0.1 μg of GFP expression vector. Eight hours post transfection, cells were untreated or treated with 1 μg/mL tunicamycin. One day later, cells were processed and cell-surface expression was monitored by flow cytometry as in Figure 2A; (**C**,**D**) the *y*-axis represents the number of events (cell count). MFI, mean fluorescence intensity. Representative flow cytometry histograms from three (**C**) or two (**D**) independent experiments are shown.

As shown in Figure 1, glycosylation of at least one Asn is required for tetherin-mediated inhibition of HIV-1 release. To determine whether complex-type oligosaccharide modification of tetherin that is glycosylated on a single residue is also dispensable for the inhibition of virus release, 293T cells were transfected with pNL4-3delVpu, and WT and tetherin mutants (N65A, N92A, and N65,92A), treated with kifunensine for 24 h, and virus release was monitored. As observed for WT tetherin, the inhibition of HIV-1 release by single Asn mutants was not diminished in the presence of kifunensine (Figure 5A,B). As expected, kifunensine treatment resulted in the expression of 23-kDa and 24.5-kDa tetherin species, but not the complex-type tetherins (≈26 to 32 kDa) for both N65A and N92A single-Asn mutants (Figure 5A). These results indicate that complex-type glycosylation of even the single-Asn mutants is dispensable for inhibition of virus release. We also tested whether preventing the complex-type glycosylation of single Asn has any effect on the surface expression of tetherin mutants. 293T cells were transfected with WT, N65A, N92A, and N65,92A mutant tetherin expression vectors that carry an HA-tag in the extracellular CC domain. Transfected cells were stained with anti-HA antibody. Although the median fluorescence intensity of cell-surface tetherin for WT and the single glycosylation mutants was slightly reduced by kifunensine treatment, there still remained a considerable amount of cell-surface tetherin following kifunensine treatment (Figure 5C). However, when WT tetherin-expressing 293T cells were treated with tunicamycin there was a marked reduction in the cell-surface expression of tetherin, suggesting that high-mannose glycosylation of tetherin, but not complex-type glycosylation, is required for cell-surface expression (Figure 5D). These results confirm our finding that complex-type glycosylation of tetherin is dispensable for cell-surface expression and inhibition of HIV-1 release, and demonstrate that a single high-mannose modification of tetherin is sufficient for cell-surface tetherin expression and antiviral activity.

## 4. Discussion

The unique topology of tetherin allows it to bind and restrict the release of virions from the surface of infected cells. Several structural domains of tetherin, including the CT, TM domain, GPI anchor, and dimerization motif in the CC region of the ectodomain, are critical for tetherin’s antiviral activity [1,2,15,20,30]. However, the role of *N*-linked glycosylation in tetherin function remains poorly defined. In this study, we carried out a detailed investigation on the role of *N*-linked glycosylation in the antiviral activity of human tetherin by using not only non-glycosylated tetherin mutants but also chemical inhibitors of the glycosylation pathway.

Our data indicate that *N*-linked glycosylation of at least one Asn residue in tetherin is required for inhibition of HIV-1 release, as mutating a single site of *N*-linked glycosylation (N65 or N92) has no impact on the antiviral activity of human tetherin. These single mutants are efficiently expressed on the cell surface, suggesting that *N*-linked glycosylation at a single Asn is sufficient for trafficking to the PM. In contrast, the double mutant, N65,92A, is defective in both cell-surface expression and inhibition of HIV-1 release. This loss of cell-surface expression and antiviral activity could not be rescued by increasing the total expression of the N65,92A mutant. Consistent with our data, Perez-Caballero et al. showed that *N*-linked glycosylation of tetherin is essential for its cell-surface expression [15]. Other labs have also reported that *N*-linked glycosylation is essential for inhibition of HIV-1 release [49,50,51,52,53,54]. Tokarev et al. observed that mutating Asn residues 65 and 92 markedly impaired the ability of tetherin to restrict virus release and induce NF-κB activity [53]. The loss of antiviral activity of the N65,92A mutant towards other viruses such as xenotropic murine leukemia virus-related virus (XMRV) was also reported [51]. Taylor et al. demonstrated that human tetherin restricts SARS-CoV egress and SARS-CoV protein ORF7a directly interacts with tetherin to inhibit its glycosylation and antiviral function [57]. It was observed that the N65,92A tetherin mutant is mis-trafficked and accumulates in CD63-positive intracellular compartments [50]. Interestingly, the non-glycosylated N65,92A tetherin mutant was still able to inhibit the release of an HIV-1 matrix mutant, 29KE/31KE, that assembles in multi-vesicular bodies (MVBs) [50,67] and also inhibited the MVB-associated hepatitis B virus (HBV) [50]. A requirement for *N*-linked glycosylation in the antiviral activity of feline tetherin was also reported [49,54]. A slight impairment in the antiviral activity of bovine tetherin was observed upon mutation of its single glycosylation site [68]. Tetherin from an owl monkey kidney cell line was shown to not restrict HIV-1 due to the presence of a Thr at residue 181 that prevented efficient glycosylation; mutation of Thr 181 to Ile rescued its glycosylation and antiviral function [69]. These studies are consistent with the importance of *N*-linked glycosylation in tetherin activity. In contrast, several other groups reported that glycosylation of human tetherin is not essential for restricting the release of HIV-1 [20], foamy virus [56], Lassa, and Marburg viruses [55]. The differences in the observations among different groups are likely due to differences in the levels of tetherin expression or the use of different approaches to measure virus release.

The studies discussed above were carried out with Asn mutants of tetherin that are defective in glycosylation. In contrast to previous studies, we also adopted the complementary approach of using chemical inhibitors of the glycosylation pathway. Treating cells with tunicamycin, which inhibits the first step of *N*-linked glycosylation, blocked the glycosylation, cell-surface expression, and antiviral activity of tetherin in a manner similar to the N65,92A double mutant. We also evaluated whether complex glycosylation of tetherin is essential for its antiviral activity. Treating cells with the mannosidase I inhibitor kifunensine resulted in the loss of complex glycosylation of WT tetherin but no loss in its cell-surface expression or antiviral function. Similarly, the cell-surface expression and antiviral activity of the single Asn mutants, N65A and N92A, were not diminished by kifunensine treatment. These results indicate that the mannose trimming activity of mannosidase I, which is required for conversion of high-mannose to complex oligosaccharide side chains, is not required for the cell-surface expression or antiviral activity of tetherin.

In conclusion, here we show that glycosylation of at least one Asn is essential, but complex side chain modifications are dispensable, for the cell-surface expression and antiviral activity of human tetherin. This study provides new insights into not only the number of oligosaccharide side chains required for tetherin transport and activity, but also the role of oligosaccharide modifications in these functions.
